# Turbulence model study for aerodynamic analysis of the leading edge tubercle wing for low Reynolds number flows

**DOI:** 10.1016/j.heliyon.2024.e32148

**Published:** 2024-05-30

**Authors:** Intizar Ali, Tanweer Hussain, Imran Nazir Unar, Laveet Kumar, Inam Ul Ahad

**Affiliations:** a Department of Mechanical Engineering, Mehran University of Engineering and Technology, Jamshoro, 76080, Sindh, Pakistan; b I-Form Advanced Manufacturing Research Centre, School of Mechanical and Manufacturing Engineering, Dublin City University, D09 V209, Dublin, Ireland; c National Center for Robotics &Automation-Condition Monitoring Systems, Mehran UET, Jamshoro, Pakistan; d Department of Chemical Engineering, Mehran University of Engineering and Technology, Jamshoro, 76080, Sindh, Pakistan; e Department of Mechanical & Industrial Engineering, College of Engineering, Qatar University, Doha, Qatar

**Keywords:** Passive flow control, Leading edge tubercle, CFD simulation, Turbulence model, Low Reynolds number flows

## Abstract

A turbulence model study was performed to analyze the flow around the Tubercle Leading Edge (TLE) wing. Five turbulence models were selected to evaluate aerodynamic force coefficients and flow mechanism by comparing with existing literature results. The selected models are realizable *k-ε*, *k-ω* Shear Stress Transport (SST), ($\gamma -Re_{\theta }$) SST model, Transition *k-k*_*l*_*-ω* model and Stress- ω Reynolds Stress Model (RSM). For that purpose, the TLE wing model was developed by using the NACA0021 airfoil profile. The wing model is designed with tubercle wavelength of 0.11c and amplitude of 0.03c. Numerical simulation was performed at chord-based Reynolds number of Re_c_ = 120,000. The Computational Fluid Dynamic (CFD) simulation reveals that among the selected turbulence models, Stress- ω RSM estimated aerodynamic forces (i.e. lift and drag) coefficients closest to that of the experimental values followed by realizable *k-ε*, ($\gamma -Re_{\theta }$) SST model, *k-ω* SST model and *k-k*_*l*_*-ω* model. However, at a higher angle of attacks i.e. at 16° & 20° *k-ω* SST model predicted closest drag and lift coefficient to that of the experimental values. Additionally, the critical observation of pressure contour confirmed that at the lower angle of attack Stress- ω RSM predicted strong Leading Edge (LE) suction followed by realizable *k-ε*, ($\gamma -Re_{\theta }$)SST model, *k-ω* SST model and *k-k*_*l*_*-ω* model. Thus, the superiority of Stress- ω RSM in predicting the aerodynamic force coefficients is shown by the flow behavior. In addition to this pressure contours also confirmed that *k-k*_*l*_*-ω* model failed to predict tubercled wing aerodynamic performance. At higher angles of attacks *k-ω* SST model estimated aerodynamic force coefficients closest to that of the experimental values, thus *k-ω* SST model is used at 16° & 20° AoAs. The observed streamline behavior for different turbulence models showed that the Stress- ω RSM model and *k-k*_*l*_*-ω* model failed to model flow behavior at higher AoAs, whereas *k-ω* SST model is a better approach to model separated flows that experience strong flow recirculation zone.

## 1 Introduction

The accurate computation of aerodynamic forces on the airfoil/wing is highly important at the design stage of wind turbines, propellers, helicopters, aircrafts and compressors. Reynolds Averaged Navier Stokes (RANS) approach based on CFD has tremendously progressed over last few decades in terms of effectiveness, robustness, and capabilities in analysing the flow field around the complex aerodynamics configuration [1,2]. However, the complexity of analysing the flow field around the aerodynamic body is significantly affected by the Reynolds number, angle of incidence and shape of the aerodynamic body due to the occurrence of different flow mechanisms such as laminar separation bubbles, transition from laminar to turbulent, flow separation and vortex shedding [[3], [4], [5], [6]]. In this regard, the accurate and detailed flow analysis under this circumstance is still a challenging issue in terms of geometric complexity, Reynolds number, and turbulences present within the flow. Moreover, in case of high Reynolds number (Re > 5 × 10^5^) flows aerodynamics is well established but the situation becomes complex when Re < 5 × 10^5^.

The Geometric complexities are enhanced through use of various flow control techniques such as flaps, slots, blowing and suction, gurney flaps, vortex generator, and leading and trailing edge modification significantly changing fluid flow behavior. The LE protuberance is the passive flow control technique employed to improve airfoil performance at high angles of attack. The unique LE tubercles or proturbance impact was first observed and analyzed by marine biologists while studying aquatic animals, Humpback whale during the 1990s [[7], [8], [9]]. The study results revealed that the exceptional design of the humpback whale flippers with protuberance LE, enables it to maintain a higher lift coefficient at higher angles of attack by allowing flow to remain attached, thus resulting delay in stall [9,10]. Thus the humpback whale is capable of performing tight turning maneuvers as a part of its feeding cycle [[11], [12], [13]]. The radius of such turns is inversely proportional to the amount of lift generated therefore any potential increment in lift is favorable [14]. Thus the morphological structure of humpback whale is beneficial in delaying stall and lowering the energy required for swimming and achieving forward velocity. This passive modification to the LE results in delaying stall and enhanced lift gaining the intention of researchers to employ it on various engineering applications. Meanwhile, numerous research studies have been conducted to employ tubercles at the LE, as a means of passive flow control to improve the aerodynamic and hydrodynamic performance of the aircraft wings [[15], [16], [17], [18]], wind turbine blades [[19], [20], [21], [22]], propellers [23], rudders [24] and hydrofoils [25]. Despite numerous applications of tubercles, still limited studies have thoroughly investigated suitable turbulence models for the accurate prediction of aerodynamic force coefficient and detailed flow analysis. Most of the research studies were conducted to analyze the aerodynamic performance of the TLE wing by using *k-ε* model [[26], [27], [28], [29]], *k-ω* SST model [[30], [31], [32], [33], [34], [35]], and Spalart Allmaras turbulence model [[36], [37], [38]]. Recently, a numerical study was performed to analyze the aerodynamic performance of the rectangular wing with waviness along the wing span at Re_c_ = 120,000. The study employed realizable *k-ε* and *k-ω* SST models for turbulence modeling, it was found that the realizable *k-ε* model predicted closer aerodynamic force coefficients in the pre-stall regime whereas it overestimated lift and drag coefficient in the post-stall regime. Moreover, *k-ω* SST model underestimates the aerodynamic force coefficient in pre-stall and accurately predicts force coefficients in post-stall. The study suggested employing realizable *k-ε* model in the pre-stall and *k-ω* SST model in the post-stall flow regime [[39], [40], [41]]. Shi, Atlar et al. [42] performed the experimental and numerical study by employing realizable *k-ε* and *k-ω* SST model and compared results with experimental results for tidal turbine blades with airfoil S814. Study results found that the *k-ω* SST model predicted closer lift and drag coefficients to that of the experimental values. Another study was conducted to analyze aerodynamic performance of full-span tubercle wing in transitional and turbulent flow regime. Study also investigated impact of Reynolds number through selecting two different Reynolds numbers 120,000 and 1,500,000. Study employed ($\gamma -Re_{\theta }$) SST model to analyze flow characteristics around rectangular wing plan-form. Study results found that in transitional flow regime baseline airfoil experience sudden loss in lift, whereas in turbulent flow regime baseline airfoil demonstrate gradual stall and produced higher lift as compared to tubercled airfoil [43]. Moreover few other studies employed ($\gamma -Re_{\theta }$) SST model to analyze the aerodynamic performance of tubercle wing at transitional flow regime with (*Re*) ranging from 100,000 to 120,000, and found good agreement with experimental results [[44], [45], [46], [47]]. Additionally, *k-ω* SST model is highly suitable for numerous industrial application flows and ($\gamma -Re_{\theta }$)SST model is also based on *k-ω* SST with addition of two-equations for considering the transition effects, in this regards to model transitional flow numerous studies found that ($\gamma -Re_{\theta }$)SST model has superior performance than *k-ω* SST model [45,46,48,49]. To assess the accuracy of turbulence models for the prediction of the lift and drag coefficient of the TLE wing only two studies have been carried out, and these studies have tested Spalart Allmaras and *k-ω* SST model only [50,51]. These studies' results found that the *S-A* model predicted aerodynamic force coefficients accurately in the pre-stall flow regime whereas it failed to predict lift and drag coefficients closer to that of experimental results in the post-stall regime. Studies suggested employing *k-ω* SST model for accurate prediction of aerodynamic force coefficients and flowing mechanism. Moreover, some studies suggested that RSM has shown close agreement with experimental results over smooth LE wing both in terms of aerodynamic forces coefficients and also in flow field prediction in pre-stall regime [52,53].

Based on above discussed literature findings it is found that most of the studies either employed a single turbulence model or only two turbulence models. However, the geometric complexity associated with LE modification in the TLE wing significantly enhances the flow complexity such as the production of the counter-rotating vortices, spanwise pressure gradient, channeling effect in the flow, and flow separation control behind the tubercle peak. Thus to accurately predict aerodynamic force coefficients and analyze flow mechanism; solving RANS equation with suitable turbulence requires further detailed analysis. In this connection, the present study assesses the accuracy of five different turbulent models in the prediction of lift and drag coefficient and flow behavior over TLE wing.

## 2 Computational methodology

To assess the accuracy of different turbulence models for analyzing the flow around the LE tubercle wing. Initially, the Computer Aided design (CAD) model of the wing is designed in Pro-Engineer software; once the model is imported the fluid domain is created around the wing model. Meshing is performed in ANSYS Meshing tool, and CFD modeling is carried out in FLUENT™, where the different turbulence models were employed for the accurate prediction of the aerodynamic performance of the TLE wing. The study is carried out within the range of angle of attacks from 0 to 20° with the interval of 4°.

### 2.1 Geometry description and meshing

**Fig. 1:**
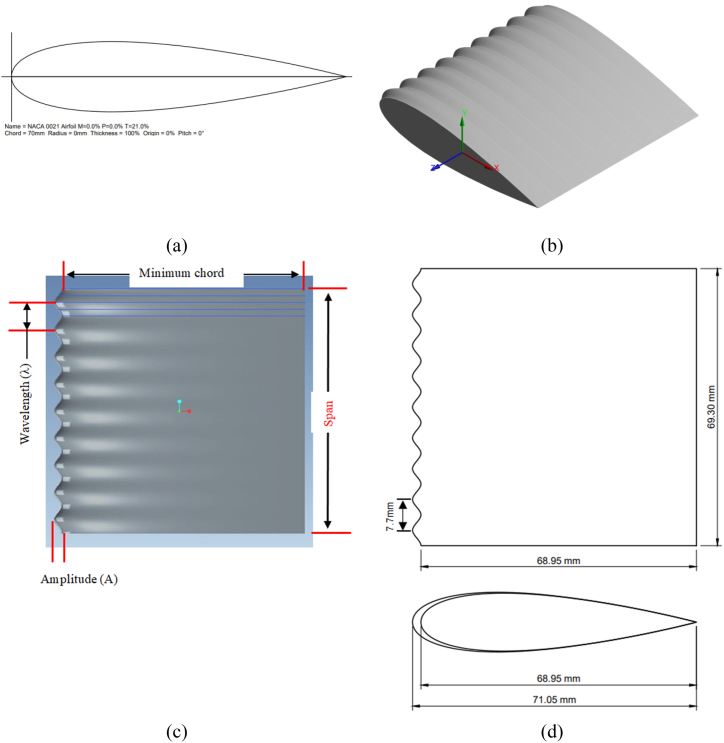
The designed sinusoidal TLE wing model (a) NACA0021 airfoil profile in 2D view with chord length of c = 70 mm (b) the isometric view of sinusoidal TLE wing model (i.e *A3λ11*), (c) top view of the *A3λ11* wing model showing amplitude (*A*), wavelength (*λ*) and span (d) Top view and front view of the designed wing model with detail dimensions at tubercle crest/peak and tubercle trough/valley.

To maintain research consistency, a uniform sinusoidal LE tubercle wing model has been generated by using the National Advisory Committee for Aeronautics (NACA) four digit symmetrical airfoil profile (i.e. NACA0021) as shown in Fig. 1(a), the same airfoil profile was used by various numerical and experimental studies [[54], [55], [56], [57], [58]]. Hansen also used the NACA0021 airfoil profile because the humpback flipper has approximately 21 % of the maximum thickness at 40 % of its chord [9]. The mean chord of the designed wing model is 70 mm and the span length is 69.3 mm (i.e. 9 wavelengths) was generated as suggested by Ref. [55] while studying the effect of wing span, and found that low wing span results in an incorrect flow pattern when span length is not sufficient. The uniform sinusoidal wing model is the optimized model among the tested models of the Hansen; according to their results sinusoidal wing model with Amplitude *A=3 %* of mean chord (*c*) and wavelength *λ=11 %* of *c* has shown better aerodynamic efficiency among tested wing models. Whereas the amplitude in this case is defined distance from tubercle valley/trough to its peak/crest, and wavelength is the distance from one peak/crest to adjacent peak/crest. According to Hansen designed parameters such as *c=*70 mm, the amplitude should be *A=2.*1 mm and wavelength value is *λ=7.*7 mm; However, Hansen considered and designed wing with amplitude of 2 mm and wavelength of 7.5 mm. Thus Hansen named his model as *A2λ7.5*, whereas in present study uniform sinusoidal tubercle wing models were designed with amplitude *A=2.*1 mm and wavelength of *λ=7.*7 mm*.* Moreover, in this research work model is named according to percentage of amplitude and wavelength thus named as (*A3λ11*). Moreover, the wing LE profile is modified along the *z* axis (spanwise direction) to developed TLE wing. The 3D CAD model is generated in Pro-Engineer software by using the coordinate transformation equation (1).

(1) $$x=c+A\;\sin\left[2\pi \left(\frac{z}{\lambda }-\frac{\lambda }{2c}\right)\right]$$

The sinusoidal TLE wing model presented in Fig. 1(b–d) is produced through employing above Eq. (1), where *x* represents the chord length at different locations along the span, *c* chord length of the wing, *A* and *λ* denotes tubercles amplitude and wavelength respectively. Where the *z* is spanwise direction where the profile of the wing is modified to generate sinusoidal tubercles at the LE. As the chord length varies along the wing span like at peak chord length is sum of mean chord and half of the tubercle amplitude, whereas at the valley/trough chord length is the difference of mean chord and half of amplitude. Moreover, tubercle wing model is developed through blending airfoils of different chord lengths to generate sinusoidal TLE wing.

**Fig. 2:**
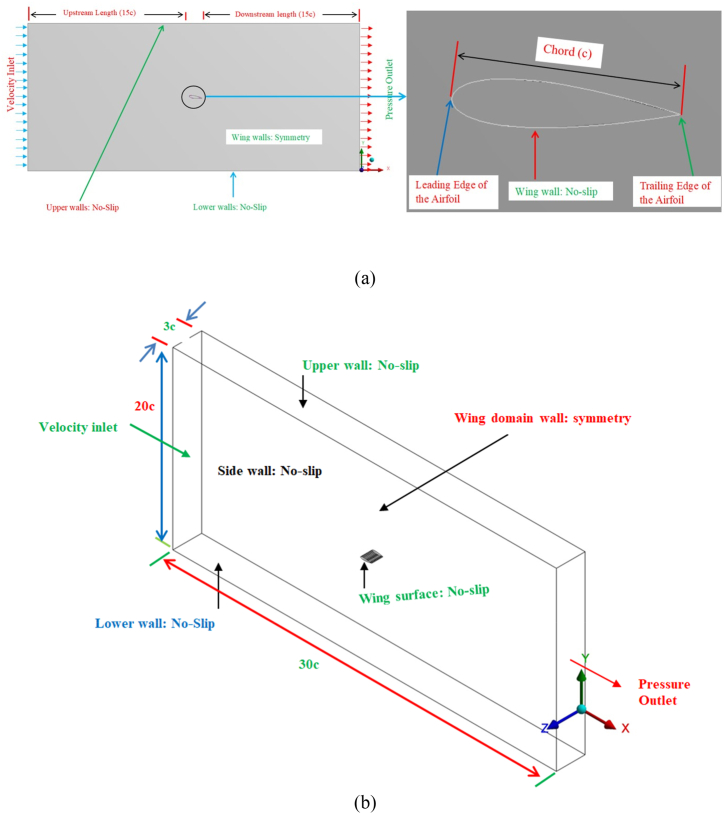
Computational domain around the tubercle wing model (a) 2D view of the domain with details of boundary conditions and zoomed view near the wing model (b)3D view of the computational domain showing dimensions of domain in terms of wing chord length and applied boundary conditions.

Once the three-dimensional (3D) wing model has been designed and developed, then the wing model has been imported to ANSYS design modeler. The imported wing model is pitched to six different angles of attacks from 0 to 20° with an interval of 4°. The fluid domain is created around the wing model and the computational domain is extended 15c (whereas c is the mean chord) in the upstream and downstream direction of the flow and 10c in the top and the bottom side of the wing models in all cases which was analogous with previous study [44]. The computational domain is then further processed to assign names to the various faces of the domain such as inlet, outlet, solid wing model and walls as shown in Fig. 2(a & b). Moreover, the 3D view of the computational domain is presented in Fig. 2(b) where the dimensions of domain are expressed in terms of the cord length of the wing.

**Fig. 3:**
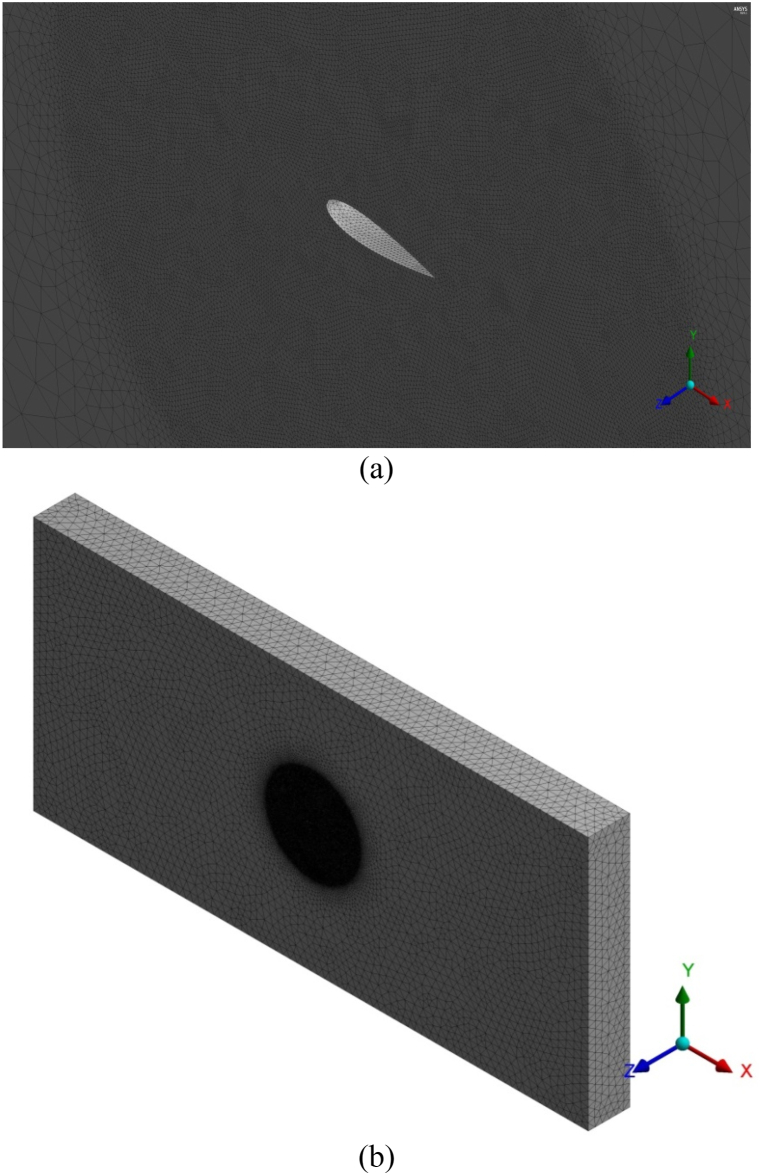
Meshed model of the fluid domain (a) Zoomed view of computational domain near the solid wing (b) meshed model of the computational domain showing the refined meshing near the wing model.

Then the meshing is carried out by using the ANSYS meshing tool, the tetrahedral mesh elements were used to generate mesh. The mesh element size throughout the fluid domain is kept at 5.00 mm except the region near the wing. The mesh size near the solid wing surface is further reduced by generating the sphere of influence; the mesh element size within the sphere of influence is kept 1.1 mm. The diameter of the sphere is set in terms of airfoil chord 6c and the size of mesh elements inside the sphere is kept at 1.1 mm. Furthermore, the growth ratio ≈1.1 is used to generate unstructured mesh around the wing model; the mesh contains the total number of cells 2.6 × 10^7^. Moreover, for predicting flow behavior accurately near the wing wall the inflation is applied in the close vicinity of the solid wing surface. The distance of the first layer from the wing surface is set at 0.053 mm, thus (Y^+^ < 1). The meshed model of the wing along with zoomed view of the mesh near the solid wing is presented in Fig. 3(a & b). The developed meshed model is then processed to solve continuity, momentum, and turbulence equations by employing Finite Volume Method (FVM) ANSYS Fluent 14.5.

### 2.2 Turbulence modeling & CFD governing equations

This research work is conducted at Re_c_ = 120,000, the corresponding freestream Mach number is quite low thus the flow is considered as incompressible. Moreover, the flow around the three-dimensional wing is considered as steady state, viscous and incompressible. The governing continuity and the RANS equations (2), (3) for steady, incompressible and viscous flow were modeled in the following manner.

(2) $$\frac{\partial U_{i}}{\partial x_{i}}=0$$

(3) $$\frac{\partial }{\partial x_{j}}\left(\rho U_{i}U_{j}\right)=-\frac{\partial P}{\partial x_{i}}+\frac{\partial }{\partial x_{j}}\left[\mu \left(\frac{\partial U_{i}}{\partial x_{j}}+\frac{\partial U_{j}}{\partial x_{i}}\right)\right]+\frac{\partial }{\partial x_{j}}\left(-\rho {\bar{u}}_{i}^{\prime }{\bar{u}}_{j}^{\prime }\right)$$

The values of *i,j=1,2,3*.

The above RANS equation is obtained through employing Reynolds averaging technique; the last term in above Eq. (3) is produced as a result of averaging technique and it is known as Reynolds stresses (RSs). This term is non-linear in nature and is modeled through employing turbulence modelling approach. Moreover, the last term of Eq. (3) is additional term that accounts for fluctuations within the mean flow terms; it is additional source of momentum. Therefore, above Eq. (3) is no longer closed, thus to model this term additional turbulence models are employed according to flow conditions. To model this term in 1877 Boussinesq first time proposed an approximation, by developing the relationship between viscous stresses and turbulent RSs. Moreover, scalar turbulent viscosity (*v*_*T*_) is used at the place of kinematic viscosity (*v*) [59]. The linear Eddy Viscosity Model (EVM) is used to model RSs by using Boussinesq approximation [59,60]. The Boussinesq approach is still used today to solve closure problems and to determine eddy viscosity (*v*_*T*_). Since the kinematic viscosity is property fluid however, the eddy viscosity is the property of flow, and it can be written as follow after employing Boussinesq hypothesis.

(4) $${\bar{u}}_{i}^{\prime }{\bar{u}}_{j}^{\prime }=\frac{2}{3}k\delta_{ij}-2v_{T}S_{ij}^{*}$$

Whereas $k=\frac{{\bar{u}}_{i}^{\prime }{\bar{u}}_{i}^{\prime }}{2}$ denotes the Turbulent Kinetic Energy (TKE), and $S_{ij}^{*}=\frac{1}{2}\left(\frac{d{\bar{u}}_{i}}{dx_{j}}+\frac{d{\bar{u}}_{j}}{dx_{i}}\right)$ the Strain tensor rate.

Moreover, Boussinesq Eq. (4) assumed that the RS tensor is proportional to mean Strain Rate (SR) *S*_*ij*_, along with that it is also assumed that turbulent diffusion is isotropic in nature. The significant accuracy of this approach is lost due to consideration of linear relationship between SR and stresses. In most of the applications of turbulent flows mostly behaves similar to that of the non-Newtonian fluid, and the viscosity depends on the SR or the history of SR, however, the linear relationship of stress with strain rate assumption is no longer valid. In this regard, the RSM is proposed to solve flow transport equations directly by using *τ*_*ij*_ and flow is assumed to be anisotropic [59].

Moreover, in comparison of the Direct Numerical simulation (DNS), Large Eddy Simulation (LES) and Detached Eddy Simulation (DES), the use of the RANS approach significantly reduces computational resources and time for practical industrial design problems. The RANS approach provides results within acceptable accuracy with the use of low gird and lower time-step size requirements. In contrast, the geometry and the flow conditions (i.e. *Re* and turbulent intensity) are of significant importance to model turbulent flows. In this regard, the TLE wing experience complex flow phenomena due to its unique leading-edge design, additionally LE tubercle airfoil is widely used in various engineering application such as compressors, turbine, propellers and aircraft wings. Thus it is highly important for accurate modeling to select appropriate turbulent model to gain reasonable relevant engineering quantities such a pressure and shear stress distribution [59]. In this connection, the present study performed fluid flow simulation of the TLE wing by employing five different turbulence models among them four models rely on the EVM approach and one on the Stress- ω RSM. The selected turbulence models include the realizable *k-ε* model [61], k- ω SST model [62], SST model [63], *k-k*_*l*_*-ω* model [64] and Stress- ω RSM [65]. Moreover, the realizable *k-ε* model assumes that eddy viscosity can modeled through two different turbulent variables; one is TKE and the second one is the dissipation rate. Since the eddy viscosity also depends on the strain that restrict over production of the turbulence in stagnant flow. The equations below describe realizable *k-ε* model.

(5) $$\frac{\partial \left(ku_{j}\right)}{\partial x_{j}}=\frac{\partial }{\partial x_{j}}\left[\left(\nu +\frac{\nu_{t}}{\sigma_{k}}\right)\frac{\partial k}{\partial x_{j}}\right]+P-\varepsilon$$

And

(6) $$\frac{\partial \left(\varepsilon u_{j}\right)}{\partial x_{j}}=\frac{\partial }{\partial x_{j}}\left[\left(\nu +\frac{\nu_{t}}{\sigma_{\varepsilon }}\right)\frac{\partial \varepsilon }{\partial x_{j}}\right]+C_{1}S\varepsilon -C_{2}\frac{\varepsilon^{2}}{k+\sqrt{\nu \varepsilon }}$$

where

$$C_{1}=\max\left[0.43,\frac{\eta }{\eta +5}\right]$$

$$\eta =S\frac{k}{\varepsilon }$$

$$S=\sqrt{2S_{ij}S_{ij}}$$

In Equation (5 & 6), P denotes the production of TKE due to the mean velocity gradients. Whereas, the turbulent Prandtl Number (P_r_) for k and ε is represented by $\sigma_{k}$ and $\sigma_{\varepsilon }$ respectively. The model constants being used present work are $C_{2}$ = 1.9, $\sigma_{k}$ = 1, and $\sigma_{\varepsilon }$ = 1.2. The *k-ω* SST model is developed, that combine the merits of k*-ω* standard model and standard *k-ε* model to accurately take into account the effects of viscous sub-layer within boundary layer and to reduce its sensitiveness to the inlet freestream turbulent intensity. In addition to that *k-ω* SST model employ the restriction on the Reynolds Shear Stress Transport (RSST) to avoid over-prediction of the eddy viscosity (*v*_*T*_).

(7) $$\frac{\partial \left(ku_{i}\right)}{\partial x_{j}}=\frac{\partial }{\partial x_{j}}\left(\frac{\Gamma_{k}\partial k}{\rho \partial x_{j}}\right)+P+Y_{k}$$

(8) $$\frac{\partial \left(\omega u_{i}\right)}{\partial x_{j}}=\frac{\partial }{\partial x_{j}}\left(\frac{\Gamma_{\omega }}{\rho }\frac{\partial \omega }{\partial x_{j}}\right)+G_{\omega }-Y_{\omega }+D_{\omega }$$

In above Eqs. (7), (8), P and $G_{\omega }$ indicates the generation of the TKE because of mean velocity gradients and the production of ω. Moreover, the effective diffusivity of k and ω, were denoted by $\Gamma_{k}$ and $\Gamma_{\omega }$ respectively. The variables $Y_{\omega }$ and $Y_{k}$ denotes the dissipation of ω and k respectively, because of turbulences present within the flow. The cross diffusion term is denoted by $D_{\omega }$. The *k-k*_*l*_*-ω* turbulence model is also developed for transition flows. The onset transition of the boundary layer from laminar to turbulent flow is accurately predicted through *k-kl-ω* model in most of the scenarios. The *k-kl-ω* model is based on the three equations to model eddy viscosity (*v*_*T*_).

(9) $$\frac{Dk_{T}}{D}=P_{K_{T}}+R+R_{NAT}-\omega k_{T}-D_{T}\frac{\partial }{\partial x_{j}}\left[\left(\nu +\frac{\alpha_{T}}{\alpha_{k}}\right)\frac{\partial k_{T}}{\partial x_{j}}\right]$$

(10) $$\frac{Dk_{L}}{D_{t}}=Pk_{L}-R-R_{NAT}-D_{L}+\frac{\partial }{\partial x_{j}}\left[\nu \frac{\partial k_{L}}{\partial x_{j}}\right]$$

(11) $$\frac{D_{\omega }}{D_{t}}=C_{\omega 1}\frac{\omega }{k_{T}}P_{K_{T}}\left(\frac{C_{\omega R}}{f_{w}}-1\right)\frac{\omega }{k_{T}}\left(R+R_{NAT}\right)-C\omega_{2}\omega^{2}+C\omega_{3}f_{\omega }\alpha_{T}f_{w}^{2}\frac{\sqrt{k_{T}}}{d^{3}}+\frac{\partial }{\partial x_{j}}\left[\left(\nu +\frac{\alpha_{\gamma }}{\alpha_{\omega }}\right)\frac{\partial \omega }{\partial x_{j}}\right]$$

In Eqs. (9), (10), (11), the terms $k_{T}$ and $k_{L}$ are used to model TKE and Laminar Kinetic Energy (LKE) of the flow respectively. Moreover, the energy related to the Tollmien-Schlichting instabilities within laminar to turbulent flow regime is denoted by $k_{L}$. The Inverse Time Scale (ITS) is denoted (ω) is modeled as ε = Kω. Moreover, the ITS capture the adverse pressure gradient accurately. It also reduces the intermittency impacts within the outer turbulent boundary layer.

The ($\gamma -Re_{\theta }$) SST model is developed to model the transition effects induced by flow variables. Moreover, it differ from the *k-ω* SST model in that, the SST model solves additional PDEs to encounter the effects of onset transition, these equations are the onset transition momentum thickness Reynolds number $R{\bar{e}}_{\theta t}$ and the intermittency (γ) equation. In order to, resolve flow through ($\gamma -Re_{\theta }$) SST model following equations (12), (13), (14), (15) were solved.

(12) $$\frac{\partial \left(\rho \gamma \right)}{\partial t}+\frac{\partial \rho U_{j}\gamma }{\partial x_{j}}=P_{x}\gamma 1-E_{\gamma 1}+P_{\gamma 2}-E_{\gamma 2}+\frac{\partial }{\partial x_{j}}\left[\left(\mu +\frac{\mu_{t}}{\sigma_{\gamma }}\right)\frac{\partial \gamma }{\partial x_{j}}\right]$$

(13) $$\frac{\partial \left(\rho R{\bar{e}}_{\theta t}\right)}{\partial t}+\frac{\partial \left(\rho U_{j}R{\bar{e}}_{\theta t}\right)}{\partial x_{j}}=P_{\theta t}+\frac{\partial }{\partial x_{j}}\left[\sigma_{\theta t}\left(\mu +\mu_{t}\right)\frac{\partial R{\bar{e}}_{\theta t}}{\partial x_{j}}\right]$$

(14) $$\frac{\partial }{\partial t}\left(\rho k\right)+\frac{\partial }{\partial x_{i}}\left(\rho ku_{i}\right)=\frac{\partial }{\partial x_{j}}\left(\Gamma_{k}\frac{\partial k}{\partial x_{j}}\right)+G_{k}^{*}-Y_{k}^{*}+S_{k}$$

(15) $$G_{k}^{*}=\gamma_{eff}{\bar{G}}_{k}$$

$$Y_{k}^{*}=\min\left(\max\left(\gamma_{eff},0.1\right),1.0\right)Y_{k}$$

Moreover, to capture flow transition effects through employing the SST model, the viscous sub-layer is resolved with highly fine meshing near the wing surface and keeping Y^+^ ≤ 1.

In addition to the abovementioned four turbulence models, the Stress-ω RSM model is also selected to model turbulent flow over the TLE wing. The RSM is known as the second moment-closer model is the most complicated turbulence model. That overcomes various shortcomings of Boussinesq approximation-based linear EVMs through uplifting assumption of isotropic turbulences. Stress-ω RSM closely resembles the *k-ω* model in predicting flow behavior [66], but the only difference is that Stress-ω RSM directly takes into account the effects of the turbulence production, diffusion and convection by using the stress transport equation. Stress-ω RSM employs a linear model for the estimation of the pressure-strain with the flow. The RSM solve the following equations for the mean velocities:

(16) $$\frac{\partial U_{i}}{\partial t}+\frac{\partial \left(\rho u_{i}u_{j}\right)}{\partial x_{j}}-\frac{\partial }{\partial x_{j}}\left[\mu \left(\frac{\partial U_{i}}{\partial x_{t}}+\frac{\partial U_{j}}{\partial x_{i}}\right)\right]=-\frac{\partial p^{\prime \prime }}{\partial x_{i}}-\frac{\partial \left(\rho {\bar{u}}_{i}{\bar{u}}_{j}\right)}{\partial x_{j}}+S_{M_{i}}$$

Whereas in the above Eq. (16) *p’’* and $S_{M_{i}}$ denotes the modified pressure and the sum body forces. Further, the fluctuating RS term is denoted by $-\rho {\bar{u}}_{i}{\bar{u}}_{j}$. Moreover, the RSM require large computation power and is difficult to converge but highly suitable for complex flows with rotation and swirling effect.

### 2.3 CFD solver setup

As the freestream velocity at the selected *Re* is quite low, thus the flow is assumed as incompressible and a pressure-based solver is selected. Steady RANS equations were solved in the lower angles of attack, whereas unsteady (URANS) equations were solved in post stall regime at 16° & 20° AoAs. The flow convergence criterion is based on the lift and drag coefficient, continuity, momentum and turbulence equations. The convergence criteria were set to be 0.00001 for all the above-mentioned equations variables. As the simulation is performed on chord chord-based Reynolds number of 120,000 the corresponding free stream velocity is computed as 25 m/s. The uniform free stream velocity is employed at the inlet with turbulence intensity of 0.8 %, by keeping velocity vectors normal to the inlet wall with direction cosine of (1, 0, 0) [67]. The no-slip condition is employed on the wing surface and on the spanwise direction walls. A zero pressure condition is employed at the outlet of the fluid domain. Moreover, the no-slip wall condition is applied on the lateral boundaries.

## 3 Results & discussion

### 3.1 Aerodynamic forces

#### 3.1.1 Lift coefficient

The aerodynamic forces such as lift, drag and pitch moment are produced as results of pressure and shear stress distribution over the wing planform. Since these forces are dimension i.e size dependent thus wing aerodynamic performance is usually expressed in terms of non-dimensional parameters like lift coefficient, drag coefficient and moment coefficient. Moreover, the lift is produced as results of pressure difference between the lower and upper surface of the wing. Once the CFD simulation is performed lift force can determined, moreover the lift coefficient (C_L_) is computed by using following relations.

(17) $$C_{L}=\frac{F_{L}}{1/2\rho U_{\infty }^{2}bc}$$

where in the above Eq. (17), FL represents the lift force, fluid density (ρ), span length (*b*) and chord length is denotes by *c*. Similarly the drag coefficient (C_D_) and is determined through following relationship.

(18) $$C_{D}=\frac{F_{D}}{1/2\rho U_{\infty }^{2}bc}$$

In Eq. (18) F_D_ denotes the drag force and other parameters has been already defined. The lift coefficient and its deviation from the experimental values are computed at different (AoAs) through employing various turbulence models has been discussed and presented in Fig. 4 (a). It can be observed from the lift coefficient versus angle of attack graph that, the lift coefficient obtained through CFD simulation is lower than experimental (C_L_) for all selected turbulence models. Further, at 0° AoA the lift coefficient of the airfoil is close to zero for all selected models because of the airfoil's symmetry. A clear difference in the predicted lift coefficient has been noticed at the remaining AoAs. Moreover, up to 8° angle of attack Stress- ω RSM and realizable *k-ε* models predicted a closer lift coefficient, however, at 12° AoA the deviation in numerical simulation C_L_ and experimental lift coefficient increased but it is within acceptable limit. The maximum deviation of 3 % is estimated in lift coefficient value as compared to the experimental value at 12° angle of attack. Moreover the, the error analysis results also showed that the Stress- ω RSM model has lowest deviation up to 12° angle of attack. Additionally, it is also noticed from the graph that among all the selected turbulence models, Stress- ω RSM has shown good agreement with the experimental lift coefficient value at a lower angle of attack. Moreover, among the selected turbulence models Stress- ω RSM model predicted lift coefficient closest to that of the experimental values followed by realizable *k-ε*, ($\gamma -Re_{\theta }$) SST model, *k-ω* SST model and *k-k*_*l*_*-ω* model. However at the higher angle of attack (i.e. 16° and 20°) *k-ω* SST model lift coefficient is closer to that of the experimental values. Moreover, in post-stall regime maximum deviation of about 1 % is estimated for *k-ω* SST model, whereas at 20° angle of attack at 20° AoA, Stress- ω RSM, realizable *k-ε* and SST model overestimated lift coefficient as compared to the experimental lift coefficient value. Furthermore, the *k-k*_*l*_*-ω* model predicted lift coefficient much lower than that of the experimental value.

**Fig. 4(a):**
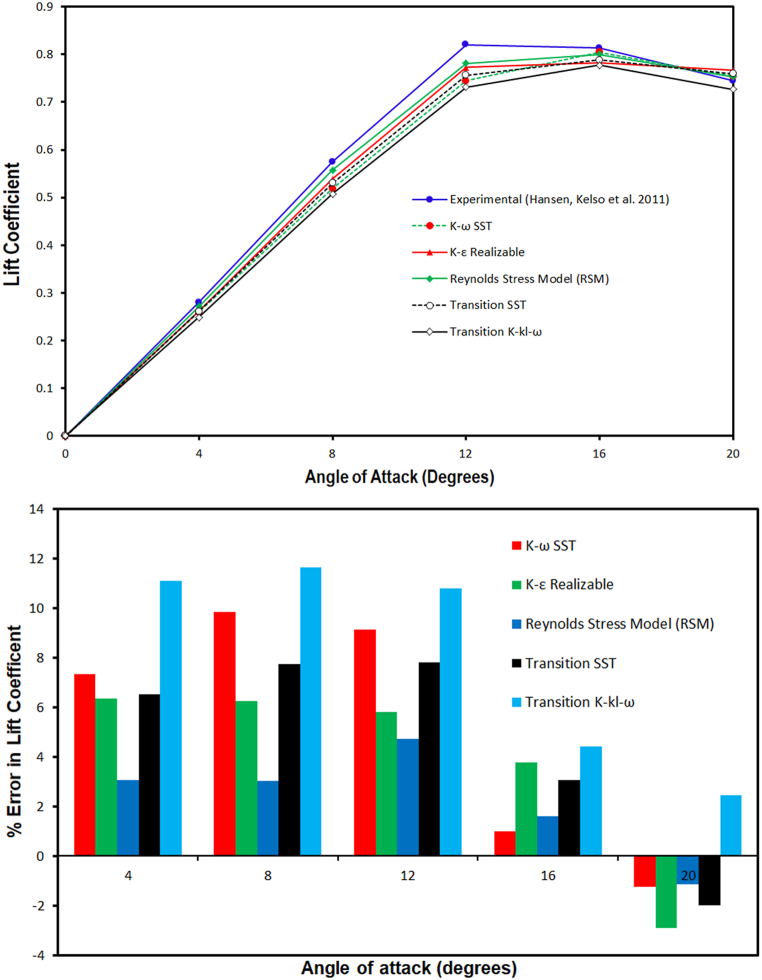
Lift coefficient values computed through CFD simulation comparison with experimental results for sinusoidal tubercled wing model Hansen, Kelso et al. (2011) [54] along with error analysis.

#### 3.1.2 Drag coefficient

The drag coefficient at the selected angle of attacks predicted through applying various turbulence models is discussed and presented in Fig. 4(b). The critical observation of drag coefficient versus angle of attack graph revealed that some turbulence models predict higher and some models predict lower drag coefficient than that of the experimental values. It is observed from the graph that, at 4° AoA drag coefficient predicted by the SST model and the Transition K-kl- ω model is higher than the experimental value. Whereas, the drag coefficient predicted through Stress-ω RSM, realizable *k-ε* and *k-ω* SST turbulence models have a close agreement with the experimental value of the drag coefficient. Moreover, among the selected turbulence models Stress-ω RSM model predicted the drag coefficient closest to the experimental value followed by realizable *k-ε*, *k-k*_*l*_*-ω* model, *k-ω* SST model and SST model in the pre-stall regime. However, at a higher angle of attack *k-ω* SST model predicted a closer drag coefficient to that of the experimental value, whereas all other selected turbulence models failed to predict the drag coefficient accurately, therefore *k-ω* SST model is employed at 16° & 20° AOA. Furthermore, the drag error analysis results revealed that Stress-ω RSM predicted drag coefficient closest to that of the experimental values up to 12° angle of attack. The maximum deviation of 3.4 % is estimated in drag coefficient value as compared to the experimental value at 12° angle of attack. The maximum deviation of about 38 % is noticed in case of *k-kl-ω* model at 4° angle of attack. However, in post-stall regime maximum deviation of about 5 % is estimated for *k-ω* SST model at 20° angle of attack.

**Fig. 4(b):**
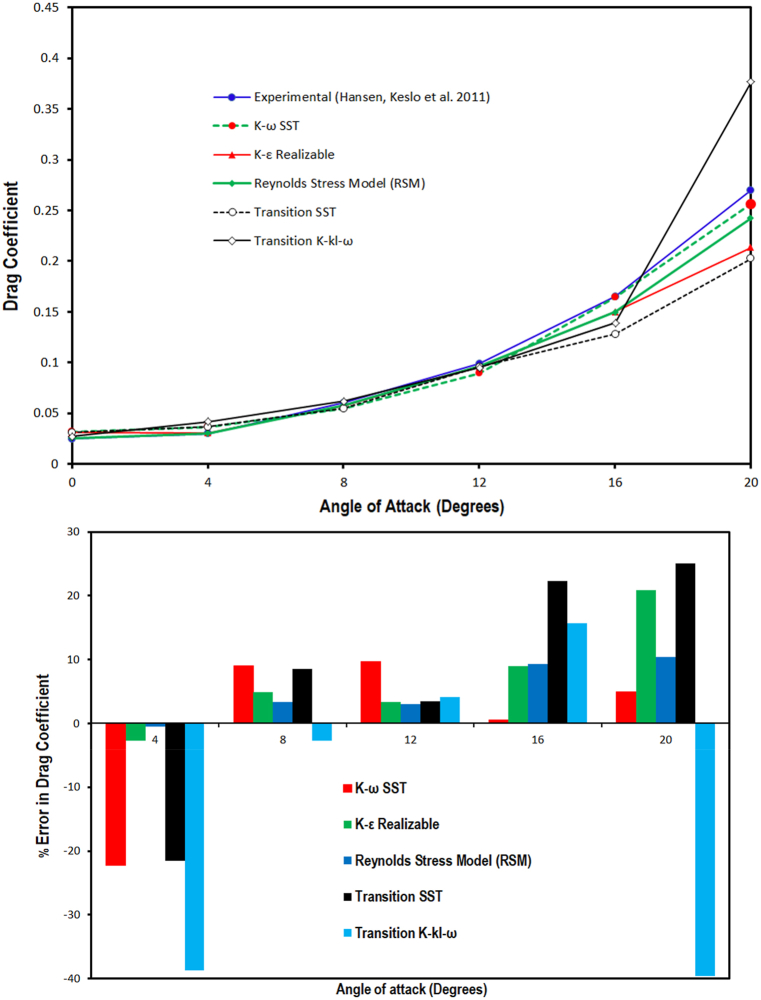
Drag coefficient values computed through CFD simulation comparison with experimental results for sinusoidal TLE wing model Hansen, Kelso et al. (2011) [54] along with error analysis.

#### 3.1.3 Lift-to-drag (L/D) ratio

The lift-to-drag ratio variation at different angles of attacks analyzed through employing various turbulence models is discussed and presented against the experimental value in Fig. 4(c). From the lift-to-drag ratio Verses angle of attack graph it is observed that all the selected turbulence models estimated lower L/D ratio within pre-stall regime than experimental values. Furthermore, among the selected models RSM model predicted the L/D ratio closest to the experimental value followed by realizable *k-ε*, ($\gamma -Re_{\theta }$) SST model, *k-ω* SST model, and *k-k*_*l*_*-ω* model. However, at a higher angle of attack all turbulence models except *k-ω* SST model failed to predict the L/D ratio accurately, therefore *k-ω* SST model is used at 16° & 20° AOA.

**Fig. 4 (c):**
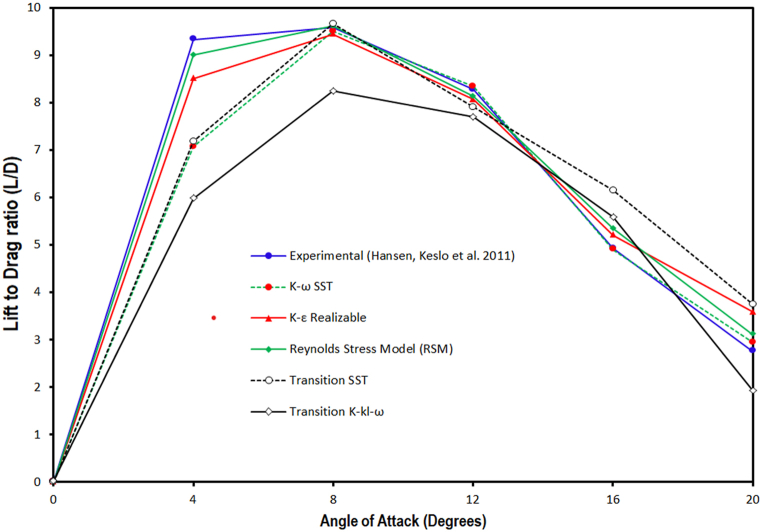
Lift-to-Drag L/D ratio values computed through CFD simulation comparison with experimental results for sinusoidal tubercled wing model Hansen, Kelso et al. (2011) [54].

### 3.2 Pressure distribution analysis

**Fig. 5:**
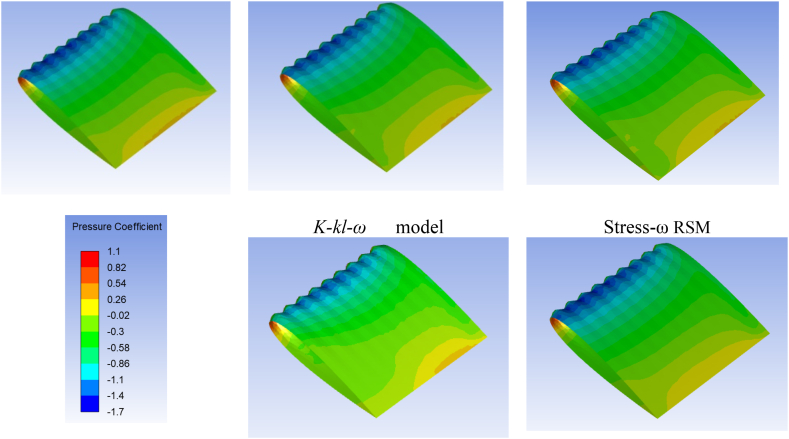
Pressure coefficient variation over the baseline and tubercled wing model for different turbulence models at 8° angle of attack.

The variation of the pressure over the wing surface obtained through employing different turbulence models is presented in Fig. 5 at 8° angle of attack. In this section, the pressure distribution over the tubercle LE wing is presented at 8° because almost similar trend of pressure variation is observed at 4° and 12° angles of attack. It is also noticed from the pressure distribution contours that pressure contours obtained through Stress-ω RSM and the realizable *k-ε* model have strong leading-edge suction. Whereas the pressure contour obtained through the *k-k*_*l*_*-ω* model shows weak LE suction at the valley and also at the peak. Moreover, from the obtained pressure contours, it is noticed that LE suction is stronger for Stress-ω RSM followed by realizable *k-ε* and *k-ω* SST model in the case of TLE wing. However, leading-edge suction in the case of the *k-k*_*l*_*-ω* model is not captured in a well manner.

**Fig. 6:**
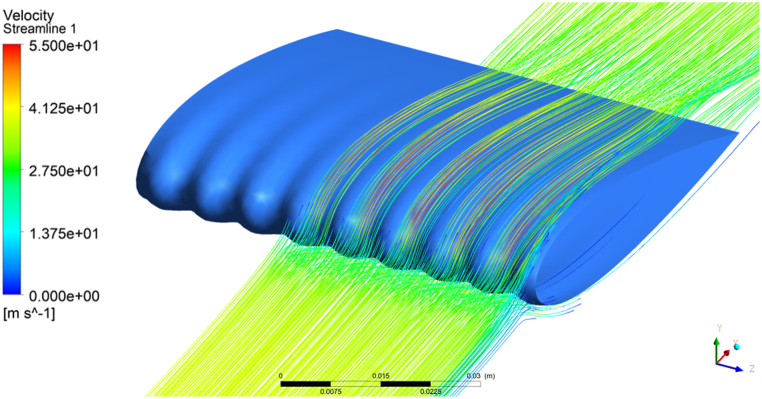
Streamline flow behaviour over the TLE wing at 8° angle of attack, showing deflection of incoming flow lines over tubercle peak.

**Fig. 7:**
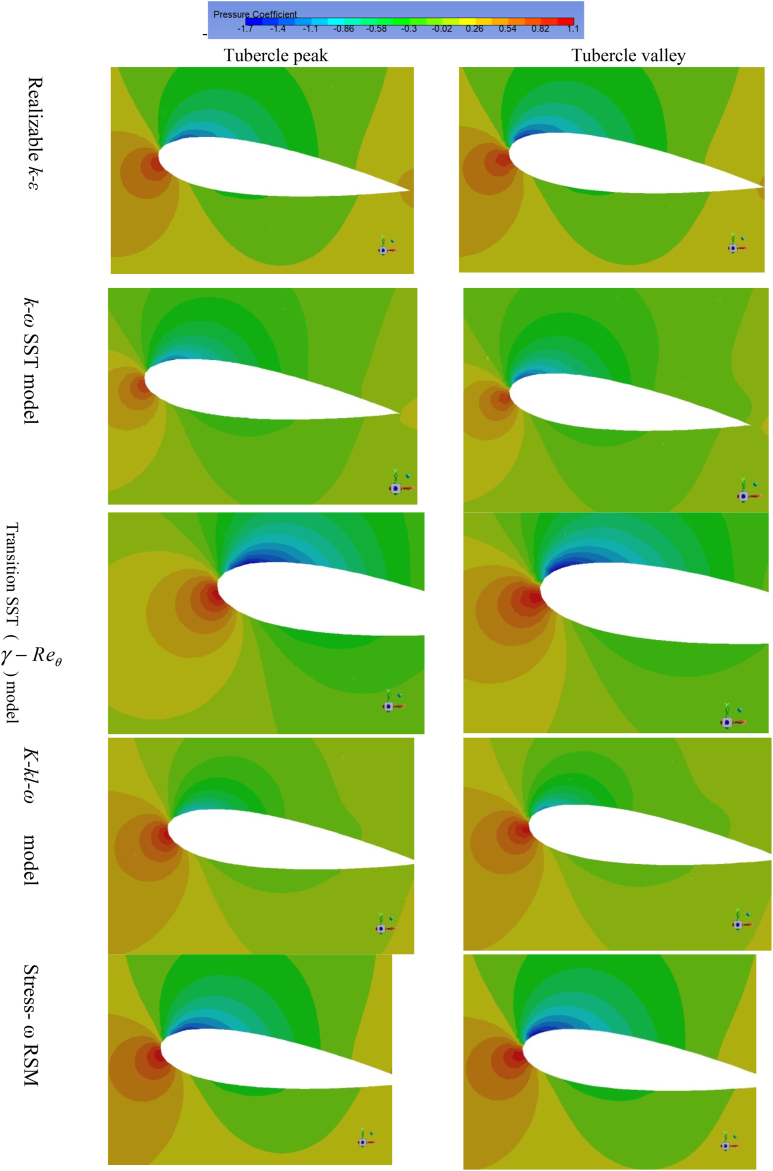
Pressure coefficient variation analyzed at tubercle peak/crest (Span location z = 11.55 mm from the fixed edge of the wing) and at tubercle trough/valley (Span location z = 7.7 mm) for different turbulence models at 8° angle of attack.

The critical analysis of the pressure distribution of the tubercle LE wing model reveals that strong leading-edge suction (i.e. negative pressure) is observed at the trough of the tubercle whereas a significant reduction in leading-edge suction is noticed at the tubercle peak. The airflow is converged by the two crests resulting in spanwise flow over the wing surface due to crest. Due modification of the LE the incoming flow is deflected towards trough, as a results of flow particles deflection towards region of minimum chord, causes enhanced suction at the trough relative to the peak of the tubercle. The results span-wise pressure gradient is apparent in in Fig. 5. It is also noticed that flow is accelerated at the trough (valley) region and causing the strong LE suction. In order to demonstrate span-wise pressure gradient in clear way streamline flow over the tubercle wing model at 8° angle of attack for Stress-ω RSM is presented in Fig. 6. Moreover, the span-wise pressure gradient is also evident from the pressure contours presented at tubercle peak and trough in Fig. 7. The reduction in tubercled wing aerodynamic performance in the pre-stall regime is mainly due to a reduction in the leading-edge suction area, because in the case of tubercled wing LE suction only at the trough region of the model, the similar observation regarding the flow behavior is noticed [39,40,44,[68], [69], [70]].

The pressure distribution/pressure contour at the tubercle peak (Span location z = 11.55 mm from the fixed edge of the wing) and at the tubercle trough (Span location z = 7.7 mm) for five selected turbulence models is discussed and presented in Fig. 7. The pressure contour is presented for selected turbulence models at 8° angle of attack near the LE of the airfoil, from the contours it is noticed that Stress- ω RSM predicted strong LE suction followed by realizable *k-ε*, ($\gamma -Re_{\theta }$) SST model, *k-ω* SST model and *k-k*_*l*_*-ω* model. Thus, pressure contours further confirm the aerodynamic forces behavior, which showed that RSM predicts tubercled wing aerodynamic performance closest to experimental values. In addition to this pressure contours also confirmed that the *k-k*_*l*_*-ω* model failed to predict tubercled wing aerodynamic performance.

To examine the detail flow mechanism that governs aerodynamic performance of TLE wing, the vorticity field is highly helpful. The different components of vorticity in Cartesian coordinate system are streamwise (freestream flow direction), and spanwise vorticity (along the negative z-axis) and transverse coordinate (normal to the streamwise direction), mathematical formulations for all three components is given as follow.

$$\begin{array}{c} \underset{˜}{\omega }=\text{Curl}\left(\underset{˜}{u}\right)=\left(\omega ,\eta ,\zeta \right)\\ \text{Streamwise}\;\text{vorticity}=\omega \;\cos\left(\alpha \right)+\eta \;\sin\left(\alpha \right)\\ \text{Tansverse}\;\text{vorticity}=-\omega \;\sin\left(\alpha \right)+\eta \;\cos\left(\alpha \right)\\ \text{Spanwise}\;\text{vorticity}=\zeta \end{array}$$

**Fig. 8:**
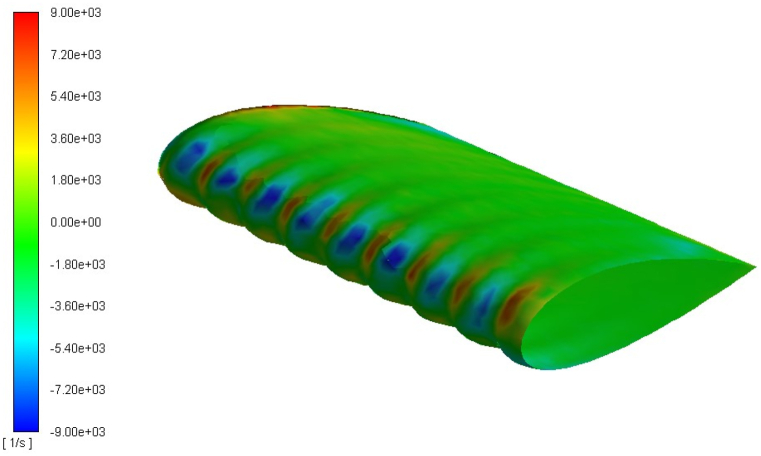
Streamwise vorticity distribution of the TLE wing at 8° angle of attack through Stress- ω RSM, while blue color shows a counter-clockwise sense of rotation, whereas red denotes a clockwise sense of rotation.

The streamwise vorticity distribution over the TLE wing is presented in Fig. 8 at 8° angle of attack, from vorticity distribution over the LE it is noticed that modification of the LE results generation of LE vortices. Moreover, opposite sign of vorticity indicates that vortices are produced in opposite direction one in clockwise direction whereas other in counter-clock wise direction. The production of pair of counter rotating vortices enhances momentum exchange within boundary layer that in turn control flow separation and delay stall. The production of pair of counter rotating vortices shown similarity that tubercles behave like vortex generator, similar flow features are noticed by various research studies [43,69,71,72].

### 3.3 Flow mechanism

**Fig. 9:**
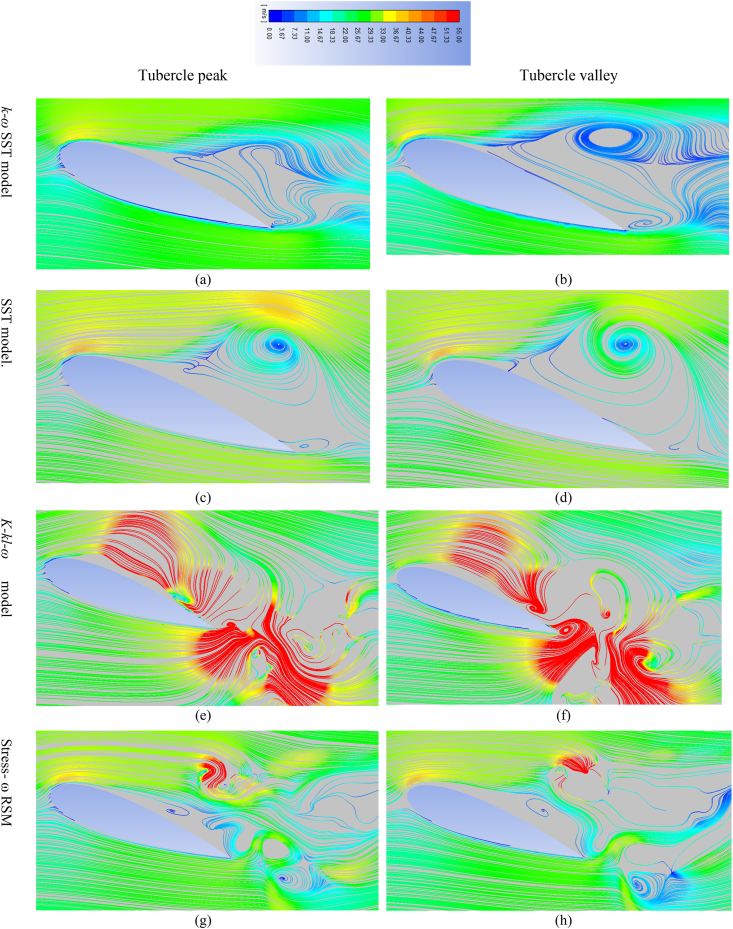
(a–h) Velocity streamline behaviour analyzed at tubercle peak/crest (Span location z = 11.55 mm from fixed edge of the wing) and at tubercle trough/valley (Span location z = 7.7 mm) for different turbulence models).

The streamline flow behavior at two different spanwise locations (i.e on the tubercle peak and the tubercle trough) at 20° angle of attack is discussed and presented in Fig. 9(a–h). The streamline flow behavior is presented for all the selected turbulence models except the k-e realizable model because it's flow behavior is close to that of the *k-ω* SST model and transition SST. From the presented streamline flow behavior it is observed that in the case *k-ω* SST model flow remains attached to the wing peak and flow separates earlier at the valley of the tubercles. Moreover, a strong flow recirculation zone is noticed above the wing surface at the tubercle valley than that of the tubercle peak. The streamline behavior at the tubercle peak and trough illustrates that flow separation is restricted behind the tubercle peak. It is also noticed that the flow recirculation zone is weak behind the tubercle peak as compared to that of the tubercle trough. These findings are in close agreement with published experimental and numerical studies [55,56,73,74]. Furthermore, ($\gamma -Re_{\theta }$) SST model turbulence model also predicted flow behavior very close to that of the *k-ω* SST model at the tubercle peak and tubercle valley region. However, the reattachment of the flow at a higher angle of attack observed in the case of the ($\gamma -Re_{\theta }$) SST model could be the reason for over predicted lift coefficient at 20° AoA. The streamline flow behavior predicted by employing the *k-k*_*l*_*-ω* model showed flow separation at the trailing edge of the airfoil only and beyond the trailing edge of the wing. Thus, the transition k-kl- ω model failed to predict flow behavior accurately at a higher angle of attack. Moreover, the streamline flow behavior obtained through Stress- ω RSM is also presented in Fig. 9(g &h). From Fig. 9(g &h) it is observed that RSM predicted stronger flow separation in the trough region as compared to that of the tubercle peak, however, RSM failed to predict flow separation closer to that of the experimental studies carried out in the past. In addition to this, it is also found that RSM also failed to accurately capture strong flow recirculation zones over the tubercle peak and valley.

## 4 Conclusion

The present study investigates the accuracy of the RANS turbulence modeling approach to predict aerodynamic force coefficients and flow mechanism against published experimental results. The study employed five different turbulence models (i.e realizable *k-ε*, *k-ω* SST model, *k-k*_*l*_*-ω* model, ($\gamma -Re_{\theta }$) SST model and Stress- ω RSM). In this regard, the fluid flow governing equations were solved over the leading tubercle wing at the chord-based Reynolds number of Re_c_ = 120,000. The study was conducted at six different angles of attacks from 0 to 20° with an interval of 4°. Based on the obtained results following conclusions were drawn.

• CFD Simulation reveals that among the selected turbulence models, Stress- ω Reynolds Stress Model (RSM) estimated lift and drag coefficient closest to that of the experimental values followed by realizable *k-ε*, ($\gamma -Re_{\theta }$) SST model, *k-ω* SST model and *k-k*_*l*_*-ω* model.
• The critical observation of pressure contour confirmed that at a lower angle of attack Stress- ω RSM predicted strong LE suction followed by realizable *k-ε*, ($\gamma -Re_{\theta }$) SST model, *k-ω* SST model and *k-k*_*l*_*-ω* model.
• Thus, the superiority of Stress- ω RSM in predicting the lift and drag coefficients closest to that of the experimental values is shown by flow behavior. In addition to this, pressure contours also confirmed that the *k-k*_*l*_*-ω* model failed to predict tubercled wing aerodynamic performance.
• In the post-stall regime i.e. at a higher angle of attack, all selected turbulence models failed to predict lift and drag coefficient accurately except *k-ω* SST, therefore *k-ω* SST model is used at 16° & 20° AOA.
• The critical observation of velocity streamline for different turbulence models showed that the Stress- ω RSM model and *k-k*_*l*_*-ω* model failed to model flow behavior at higher angles of attack, whereas *k-ω* SST model is a better approach to model separated flows which experience strong flow recirculation zone.

## CRediT authorship contribution statement

**Intizar Ali:** Writing – original draft, Validation, Software, Methodology, Investigation, Formal analysis, Data curation, Conceptualization. **Tanweer Hussain:** Writing – review & editing, Visualization, Validation, Supervision, Methodology, Formal analysis, Conceptualization. **Imran Nazir Unar:** Writing – review & editing, Validation, Supervision, Software, Methodology, Formal analysis, Conceptualization. **Laveet Kumar:** Writing – review & editing, Validation, Supervision, Investigation, Funding acquisition, Formal analysis, Methodology. **Inam Ul Ahad:** Writing – review & editing, Visualization, Supervision, Formal analysis.

## Declaration of competing interest

The authors declare that they have no know competing personal relationship or financial interests that could have appeared to influence the work reported in this paper.

## Nomenclature

k: Turbulent kinetic energy
$R{\bar{e}}_{\theta t}$: Onset transition momentum-thickness Reynolds number
c: Chord length
Re_c_: Chord-based Reynolds number
k_L_: laminar kinetic energy
*A*: Tubercle amplitude
*x*: The chord length at different span locations
*Y*^*+*^: Dimensionless wall distance
*U*_*i*_: Upstream velocity with its direction cosine
P: Freestream pressure
*B*: Span-length
C_L_: Lift coefficient
F_L_: Lift force
C_D_: Drag coefficient
F_D_: Drag force
$U_{\infty }$: Freestream velocity

**Geek symbols**
Sij: Mean strain rate
*p’’*: Modified pressure
$S_{M_{i}}$: Sum of all body force
ρ: Fluid Density
μ: Dynamic viscosity
$\nu_{T}$: Eddy viscosity
ε: Turbulent dissipation rate
ω: specific rate of dissipation
γ: Intermittency
*λ*: Tubercle wavelength
$\sigma_{k}$: Turbulent Prandtl numbers for k
$\sigma_{\varepsilon }$: Turbulent Prandtl numbers for ε
$G_{\omega }$: Generation of ω
$\Gamma_{k}$: Effective diffusivity of k
$\Gamma_{\omega }$: Effective diffusivity of ω
$Y_{k}$: Dissipation of k
$Y_{\omega }$: Dissipation of ω
$D_{\omega }$: Cross-diffusion

**Abbreviations**
SST: Shear Stress Transport
RSM: Reynolds Stress Model
TLE: Tubercle Leading Edge
LE: Leading Edge
3D: Three dimensional
Re: Reynolds number
NACA: National Advisory Committee for Aeronautics
CFD: Computational Fluid Dynamics
AoA: Angle of Attack
RANS: Reynolds-Averaged Navier–Stokes
CAD: Computer Aided design
FVM: Finite Volume Method
RSs: Reynolds stresses
SR: Strain Rate
TKE: Turbulent Kinetic Energy
DNS: Direct Numerical simulation
LES: Large Eddy Simulation
DES: Detached Eddy Simulation
PDEs: Partial Differential Equations

## References

[1] Kroll N., Schwamborn D., Becker K., Rieger H., Thiele F. (2007).

[2] Rossow C.-C., Cambier L. (2009).

[3] Castelli M.R., Garbo F., Benini E. (2011). Numerical investigation of laminar to turbulent boundary layer transition on a NACA 0012 airfoil for vertical-axis wind turbine applications. Wind Eng..

[4] Cebeci T., Platzer M., Chen H., Chang K.-C., Shao J.P. (2005).

[5] Wang S., Ingham D.B., Ma L., Pourkashanian M., Tao Z. (2010). Numerical investigations on dynamic stall of low Reynolds number flow around oscillating airfoils. Comput. Fluids.

[6] Wang S., Ingham D.B., Ma L., Pourkashanian M., Tao Z. (2012). Turbulence modeling of deep dynamic stall at relatively low Reynolds number. J. Fluid Struct..

[7] Bushnell D.M., Moore K. (1991). Drag reduction in nature. Annu. Rev. Fluid Mech..

[8] Fish F.E. (1994). Influence of hydrodynamic-design and propulsive mode on mammalian swimming energetics. Aust. J. Zool..

[9] Fish F.E., Battle J.M. (1995). Hydrodynamic design of the humpback whale flipper. J. Morphol..

[10] Miklosovic D., Murray M., Howle L., Fish F. (2004). Leading-edge tubercles delay stall on humpback whale (Megaptera novaeangliae) flippers. Phys. Fluids.

[11] Edel R., Winn H. (1978). Observations on underwater locomotion and flipper movement of the humpback whale Megaptera novaeangliae. Mar. Biol..

[12] Hw J., Carter R., Kraus D., Mayo A., Winni E. (1982). Feeding behavior of the humpback whale, Megaptera novaeangliae, in the western North Atlantic. Fish. Bull..

[13] Jurasz C.M., Jurasz V.P. (1979).

[14] Weihs D. (1981). Effects of swimming path curvature on the energetics of fish motion. Fish. Bull..

[15] Chen W., Qiao W., Wei Z. (2020). Aerodynamic performance and wake development of airfoils with wavy leading edges. Aero. Sci. Technol..

[16] Papadopoulos C., Katsiadramis V., Yakinthos K. (2019). MATEC Web of Conferences.

[17] Rohmawati I., Arai H., Nakashima T., Mutsuda H., Doi Y. (2020). Effect of wavy leading edge on pitching rectangular wing. J. Aero Aqua Bio-Mech..

[18] Zhang Y., Zhang X., Yi L., Chang M., Jiakuan X. (2021). Aerodynamic performance of a low-Reynolds UAV with leading-edge protuberances inspired by humpback whale flippers. Chin. J. Aeronaut..

[19] Yan Y., Avital E., Williams J., Cui J. (2021). Aerodynamic performance improvements of a vertical axis wind turbine by leading-edge protuberance. J. Wind Eng. Ind. Aerod..

[20] Zhang Y., Zhang M., Cai C. (2019). Flow control on wind turbine airfoil affected by the surface roughness using leading-edge protuberance. J. Renew. Sustain. Energy.

[21] Zhang Y., Zhang M., Cai C., Xu J. (2020). Aerodynamic load control on a dynamically pitching wind turbine airfoil using leading-edge protuberance method. Acta Mech. Sin..

[22] Zhao M., Xu L., Tang Z., Zhang X., Zhao B., Liu Z. (2021). Onset of dynamic stall of tubercled wings. Phys. Fluids.

[23] Weber P.W., Howle L.E., Murray M.M. (2010). Lift, drag, and cavitation onset on rudders with leading-edge tubercles. Marine Technol. Sname News.

[24] Lin C. (2009). Preliminary study on the effect of leading edge protuberances on B-series propeller's performance. MS thesis.

[25] Wei Z., Zang B., New T., Cui Y. (2016). A proper orthogonal decomposition study on the unsteady flow behaviour of a hydrofoil with leading-edge tubercles. Ocean Eng..

[26] Corsini A., Delibra G., Sheard A.G. (2013). On the role of leading-edge bumps in the control of stall onset in axial fan blades. J. Fluid Eng..

[27] Fernandes I., Sapkota Y., Mammen T., Rasheed A., Rebello C., Kim Y.H. (2013). 2013 Aviation Technology, Integration, and Operations Conference.

[28] Gawad A.F.A. (2013). Utilization of whale-inspired tubercles as a control technique to improve airfoil performance. Trans. Control Mech. syst..

[29] Isaac K., Swanson T. (2011). 6th AIAA Theoretical Fluid Mechanics Conference.

[30] Lohry M.W., Martinelli L., Kollasch J.S. (2013). 31st AIAA Applied Aerodynamics Conference.

[31] Wang Y.-y., Hu W.-r., Zhang S.-d. (2014). Performance of the bio-inspired leading edge protuberances on a static wing and a pitching wing. J. Hydrodyn..

[32] Xingwei Z., Chaoying Z., Tao Z., Wenying J. (2013). Numerical study on effect of leading‐edge tubercles. Aircraft Eng. Aero. Technol..

[33] Yoon H., Hung P., Jung J., Kim M. (2011). Effect of the wavy leading edge on hydrodynamic characteristics for flow around low aspect ratio wing. Comput. Fluid.

[34] Kim M.J., Yoon H.S., Jung J.H., Chun H.H., Park D.W. (2012). Hydrodynamic characteristics for flow around wavy wings with different wave lengths. Int. J. Nav. Archit. Ocean Eng..

[35] Fan M., Dong X., Li Z., Sun Z., Feng L. (2022). Numerical and experimental study on flow separation control of airfoils with various leading-edge tubercles. Ocean Eng..

[36] Cai C., Zuo Z., Liu S., Wu Y., Wang F. (2013). IOP Conference Series: Materials Science and Engineering.

[37] Dropkin A., Custodio D., Henoch C., Johari H. (2012). Computation of flow field around an airfoil with leading-edge protuberances. J. Aircraft.

[38] Kouh J.-S., Lin H.-T., Lin T.-Y., Yang C.-Y., Nelson B.-S. (2011). 11th International Conference on Fluid Control, Measurements and Visualization (FLUCOME 2011).

[39] Ali I., Hussain T., Unar I.N., Waqas M. (2022). Aerodynamic performance analysis of the wavy wing with varying spanwise waviness characteristics. Bull. Am. Phys. Soc..

[40] Tunio I., Kumar D., Hussain T., Jatoi M., Safiullah (2020). Investigation of variable spanwise waviness wavelength effect on wing aerodynamic performance. Fluid Dynam..

[41] Tunio I.A., Shah M.A., Hussain T., Harijan K., Mirjat N.H., Memon A.H. (2020). Investigation of duct augmented system effect on the overall performance of straight blade Darrieus hydrokinetic turbine. Renew. Energy.

[42] Shi W., Atlar M., Norman R., Aktas B., Turkmen S. (2016). Numerical optimization and experimental validation for a tidal turbine blade with leading-edge tubercles. Renew. Energy.

[43] Rostamzadeh N., Kelso R.M., Dally B. (2017). A numerical investigation into the effects of Reynolds number on the flow mechanism induced by a tubercled leading edge. Theor. Comput. Fluid Dynam..

[44] Fan M., Sun Z., Dong X., Li Z. (2022). Numerical and experimental investigation of bionic airfoils with leading-edge tubercles at a low-Re in considering stall delay. Renew. Energy.

[45] Rostamzadeh N., Kelso R., Dally B., Hansen K. (2013). The effect of undulating leading-edge modifications on NACA 0021 airfoil characteristics. Phys. Fluids.

[46] Wang H., Jiang X., Chao Y., Li Q., Li M., Zheng W. (2019). Effects of leading edge slat on flow separation and aerodynamic performance of wind turbine. Energy.

[47] Sreejith B., Sathyabhama A. (2020). Experimental and numerical study of laminar separation bubble formation on low Reynolds number airfoil with leading-edge tubercles. J. Braz. Soc. Mech. Sci. Eng..

[48] Aftab S.M., Ahmed K.A. (2019). AIAA SciTech 2019 Forum.

[49] Sørensen N.N. (2009). CFD modelling of laminar‐turbulent transition for airfoils and rotors using the γ− model. Wind Energy: An International Journal for Progress and Applications in Wind Power Conversion Technology.

[50] Lohry M.W., Clifton D., Martinelli L. (2012). Seventh InternatiAn Int. J. Prog. Appl. Wind Power Convers. Technol.onal Conference on Computational Fluid Dynamics (ICCFD7).

[51] Weber P.W., Howle L.E., Murray M.M., Miklosovic D.S. (2011). Computational evaluation of the performance of lifting surfaces with leading-edge protuberances. J. Aircraft.

[52] Apsley D., Leschziner M. (2001). Investigation of advanced turbulence models for the flow in a generic wing-body junction. Flow, Turbul. Combust..

[53] Ding Y., de Silva C., Doolan C., Moreau D. (2022). Investigation of the mean pressure field in the wing-wall junction region. Int. J. Heat Fluid Flow.

[54] Hansen K.L., Kelso R.M., Dally B.B. (2011). Performance variations of leading-edge tubercles for distinct airfoil profiles. AIAA J..

[55] Pérez-Torró R., Kim J.W. (2017). A large-eddy simulation on a deep-stalled aerofoil with a wavy leading edge. J. Fluid Mech..

[56] Skillen A., Revell A., Pinelli A., Piomelli U., Favier J. (2015). Flow over a wing with leading-edge undulations. AIAA J..

[57] Tunio I., Kumar D., Hussain T., Jatoi M. (2020). Investigation of variable spanwise waviness wavelength effect on wing aerodynamic performance. Fluid Dynam..

[58] Zhao M., Zhao Y., Liu Z., Du J. (2019). Proper orthogonal decomposition analysis of flow characteristics of an airfoil with leading edge protuberances. AIAA J..

[59] Weinmann M. (2011).

[60] Schmitt F.G. (2007). About Boussinesq's turbulent viscosity hypothesis: historical remarks and a direct evaluation of its validity. Compt. Rendus Mec..

[61] Shih T.-H., Liou W.W., Shabbir A., Yang Z., Zhu J. (1995). A new k-ϵ eddy viscosity model for high Reynolds number turbulent flows. Comput. Fluids.

[62] Menter F.R., Kuntz M., Langtry R. (2003). Ten years of industrial experience with the SST turbulence model. Turbulence, heat and mass transfer.

[63] Menter F.R., Langtry R., Völker S. (2006). Transition modelling for general purpose CFD codes. Flow, Turbul. Combust..

[64] Walters D.K., Cokljat D. (2008). A three-equation eddy-viscosity model for Reynolds-averaged Navier–Stokes simulations of transitional flow. J. Fluid Eng..

[65] Fluent A.F.T.G. (2020).

[66] Wilcox D.C. (2006).

[67] Hansen K., Kelso R., Dally B. (2010). 17th Australasian Fluid Mechanics Conference.

[68] Butt F.R., Talha T. (2019). Numerical investigation of the effect of leading-edge tubercles on propeller performance. J. Aircraft.

[69] Hansen K.L. (2012).

[70] Watts P., Fish F.E. (2001). Proc. Twelfth Intl. Symp. Unmanned Untethered Submers. Technol.

[71] Hansen K.L., Rostamzadeh N., Kelso R.M., Dally B.B. (2016). Evolution of the streamwise vortices generated between leading edge tubercles. J. Fluid Mech..

[72] Kim H., Kim J., Choi H. (2018).

[73] Hrynuk J.T., Bohl D.G. (2020). The effects of leading-edge tubercles on dynamic stall. J. Fluid Mech..

[74] Zhang Y.-N., Cao H.-J., Zhang M.-M. (2021). Investigation of leading-edge protuberances for the performance improvement of thick wind turbine airfoil1. J. Wind Eng. Ind. Aerod..

